# Efficacy of Motion Sensor Telerehabilitation for Balance and Mobility in Parkinson′s Disease: A Nonrandomized Controlled Pilot Study

**DOI:** 10.1155/ijta/7191579

**Published:** 2026-02-25

**Authors:** Issaree Prukviwat, Krisna Piravej, Viboon Sangveraphunsiri, Weerachai Jitpugdee, Pim Terachinda

**Affiliations:** ^1^ Thai Red Cross Rehabilitation Center, Thai Red Cross Society, Samut Prakan, Thailand, redcross.or.th; ^2^ Department of Rehabilitation Medicine, Faculty of Medicine, Chulalongkorn University, Bangkok, Thailand, chula.ac.th; ^3^ International School of Engineering, Faculty of Engineering, Chulalongkorn University, Bangkok, Thailand, chula.ac.th; ^4^ Department of Rehabilitation Medicine, King Chulalongkorn Memorial Hospital, Bangkok, Thailand, chulalongkornhospital.go.th

**Keywords:** gait, Parkinson′s disease, physical functional performance, rehabilitation, telerehabilitation

## Abstract

**Purpose:**

We developed an innovative telerehabilitation system using a 3D camera with motion sensors that provided real‐time feedback. This study is aimed at evaluating its efficacy in improving balance, gait, and mobility, as well as its feasibility in patients with idiopathic Parkinson′s disease (PD).

**Materials and Methods:**

Participants with idiopathic PD self‐selected into either a telerehabilitation (tele) group or a hospital‐based rehabilitation (hospital) group. The tele group received two initial sessions of hospital‐based rehabilitation sessions, followed by 14 telerehabilitation sessions using the innovative system. The hospital group received 16 sessions of hospital‐based rehabilitation. Outcome measures included Berg Balance Scale (BBS) score, Chula Parkinson Mobility Scale (Chula PMS) score, gait speed, and step length. The feasibility of the telerehabilitation system was also assessed.

**Results:**

Forty‐six participants were recruited (tele group: *n* = 23; hospital group: *n* = 23). Both groups showed statistically significant improvements in the BBS scores (tele: post–pre = 3.50, *p* < 0.001; hospital: post–pre = 4.35, *p* < 0.001), with no statistically significant difference between the groups (mean difference: −0.85, *p* = 0.454). Chula PMS score also improved significantly in both groups (tele: post–pre = 3.45, *p* < 0.001; hospital: post–pre = 5.70, *p* < 0.001) without a statistically significant difference between the groups (mean difference: −2.25, *p* = 0.086). The attendance rate exceeded 90% in both groups.

**Conclusions:**

The motion sensor telerehabilitation significantly improved balance and mobility in PD patients with no statistically significant differences between the two groups. Feasibility was high. However, the BBS improvements did not reach the minimal clinically important difference, indicating the need for further investigation.

**Trial Registration:**

Thai Clinical Trials Registry identifier: TCTR 20220924001

## 1. Introduction

Parkinson′s disease (PD) is a chronic neurodegenerative disorder. It is predicted that the number of cases will increase to 12 million by 2040 [1]. In Thailand, the prevalence of the disease was 242.57 per 100,000 people, equivalent to 1% of people over 60 years of age [2].

The cardinal symptoms of PD are tremor, bradykinesia, rigidity, and postural instability, all of which indicate disease progression [3]. These symptoms can lead to falls and fractures, increasing morbidity and mortality [4].

Among these symptoms, balance impairment is particularly disabling and has become a primary focus of rehabilitation. Studies have shown that physical therapy can significantly improve balance and gait in patients with PD [5–8]. However, substantial barriers to accessing medical services remain, including travel time, travel costs, and availability of caregivers to accompany patients to medical facilities [9]. These accessibility challenges highlight the need for technology‐supported rehabilitation models that facilitate home‐based therapy.

Furthermore, the COVID‐19 pandemic has made it difficult to access in‐hospital rehabilitation services, making telerehabilitation an excellent way to ensure accessibility for patients requiring rehabilitation. Several studies have demonstrated that telerehabilitation is comparable to standard treatments in improving functional outcomes in various conditions [10–12].

In recent years, various technologies have been applied to telerehabilitation for patients with PD, including phones, computers, applications, videos, games, motion sensors, and virtual reality, contributing to reductions in motor impairment [13]. However, no studies to date have evaluated the efficacy of telerehabilitation that incorporates real‐time feedback using a 3D camera and motion sensors compared with conventional hospital‐based rehabilitation in patients with PD.

To address this gap, we developed a motion sensor telerehabilitation system that enables supervised home‐based rehabilitation with real‐time feedback from physical therapists (PTs). The system includes a 3D camera and motion sensors to detect patients′ movements, a microphone and speaker for communication, and a display screen for exercise videos. Real‐time system feedback assists the PT in remotely correcting patients′ movements during each session.

This study is aimed at evaluating the efficacy of our telerehabilitation system in improving balance, gait, and physical performance in patients with PD, as well as to assess its feasibility for home‐based rehabilitation.

## 2. Materials and Methods

This study was approved by the Institutional Review Board of the Faculty of Medicine, Chulalongkorn University (IRB No. 0255/65). It was conducted at the Advanced Geriatric Rehabilitation Clinic of King Chulalongkorn Memorial Hospital between September 2022 and November 2023.

### 2.1. Study Design

This was a patient‐preference, nonrandomized controlled pilot study with a blinded assessor.

### 2.2. Participants

Participants were recruited from outpatient clinics at King Chulalongkorn Memorial Hospital and other hospitals. All participants were informed about the study and provided written informed consent prior to enrollment. They were allowed to choose between participating in the telerehabilitation (tele) group or the hospital‐based rehabilitation (hospital) group, which was conducted at a tertiary care center. The target sample size was 20 participants per group, with an additional 10% added to account for potential dropouts.

Inclusion criteria were as follows:

1. Diagnosed with idiopathic PD by a neurologist
2. Age ≥ 20 years
3. Modified Hoehn and Yahr scale between 1.5 and 3
4. Able to communicate effectively and follow instructions
5. Stable vital signs
6. No changes in PD medication dosage within 2 weeks prior to enrollment

Exclusion criteria were as follows:

1. Visual impairment that prevents the use of the telerehabilitation system
2. Severe musculoskeletal pain affecting outcome assessment or participation in rehabilitation
3. Other neurological disorders
4. Thai Mental State Examination (TMSE) score ≤ 23
5. Participation in a rehabilitation program within 3 months prior to enrollment

### 2.3. Interventions

#### 2.3.1. Telerehabilitation Group

Participants initially received a one‐on‐one exercise program with a PT, consisting of two 45‐min sessions focused on improving lower extremity strength and balance training. Following these sessions, participants continued the same exercise program at home, under remote supervision by a PT via a telerehabilitation system (Figure 1). They completed 14 additional sessions, each lasting 45 min, conducted twice weekly. The exercise video was displayed on the system screen, while a stereovision camera and motion sensors captured three‐dimensional body positioning and relayed data to a monitor system. This setup provided real‐time feedback to both the participants and the remotely monitoring PT.

Figure 1(a) The innovative telerehabilitation system. (b) Motion sensor detection interface. The motion sensors, trained to recognize correct postures, displayed green dots on the screen when the participant maintained proper form according to the exercise video. Red dots appeared when the participant adopted an incorrect posture.

**Figure:**
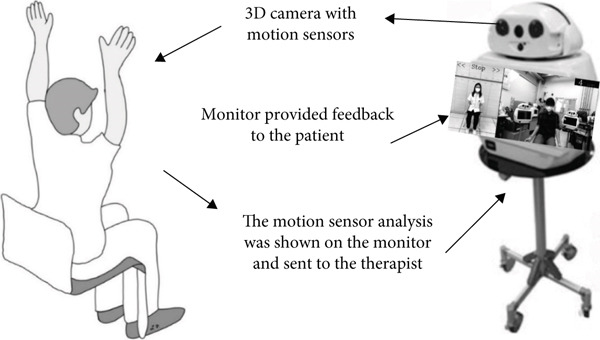
(a)

**Figure:**
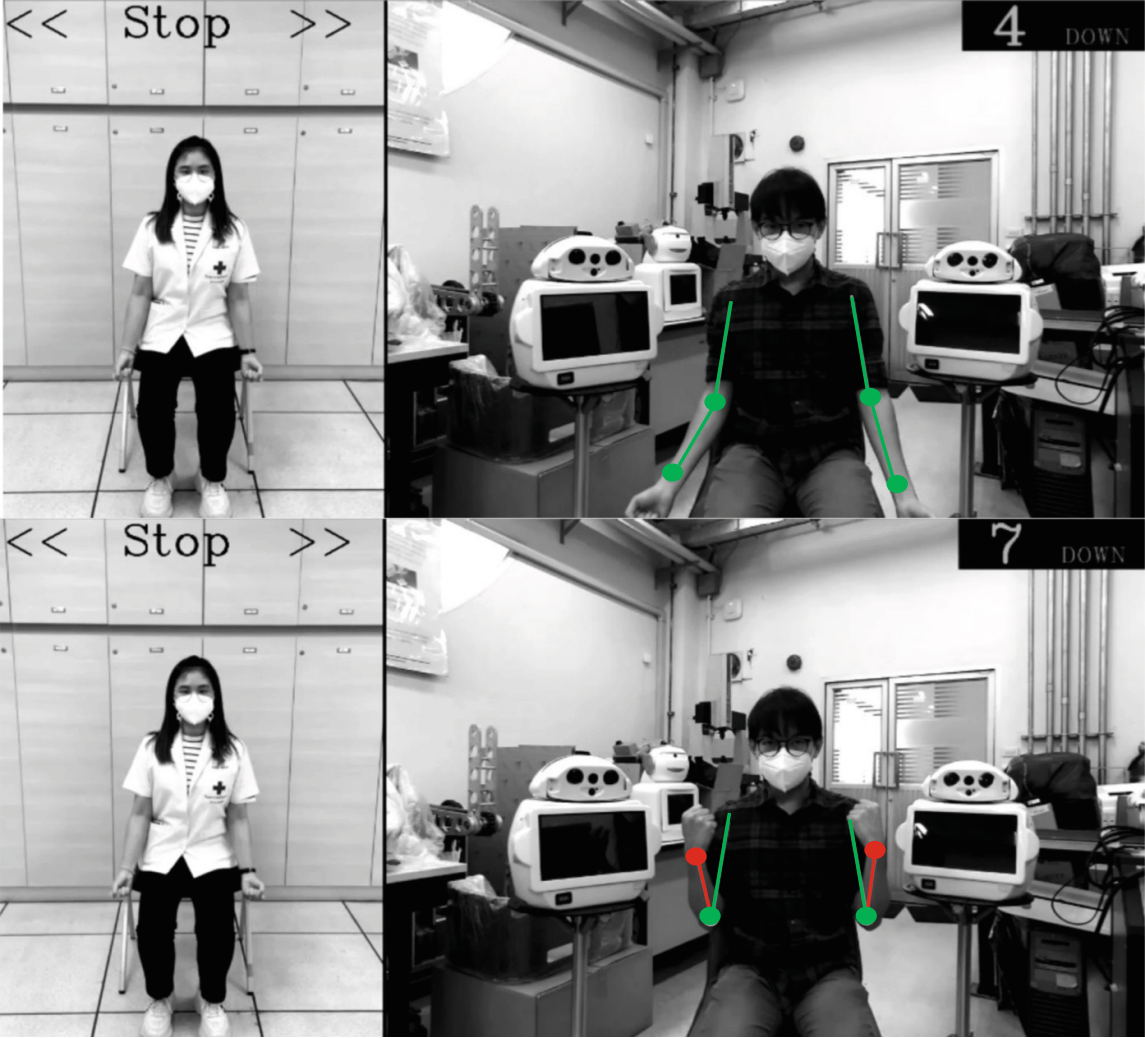
(b)

#### 2.3.2. Hospital‐Based Rehabilitation Group

Participants in this group received the same exercise program as the tele group. They engaged in 45‐min, one‐on‐one training sessions conducted at the hospital, twice a week, for a total of 16 sessions.

The exercise program consisted of 28 exercises, including strengthening exercises for the upper and lower extremities, as well as balance training. These exercises were identical for both groups.

### 2.4. Effect of Other Treatments

All participants were advised not to undergo any other rehabilitation program or begin a new exercise program during the study. Assessments and exercises were conducted while the participants were in a stable on‐medication state for their PD.

### 2.5. Dropout Criteria

Participants were classified as dropouts if one or more of the following conditions applied:

1. The participant chose to discontinue their participation in the study.
2. The participant attended less than 80% of the scheduled sessions.
3. A medical condition prevented the participant from continuing the rehabilitation program.
4. A condition interfered with the use of the telerehabilitation system in the tele group (e.g., inability to operate the device).
5. The participant underwent an adjustment of their PD medication during the study.

### 2.6. Baseline Characteristics

Baseline data collected included gender, age, modified Hoehn and Yahr scale, TMSE score, level of education, duration of symptoms, body mass index (BMI), and all baseline outcome measures.

### 2.7. Outcome Measurement

Pre‐ and posttreatment outcomes were assessed at the hospital by the same assessor, who was blinded to the participant group.

• Primary outcome
  – *Berg Balance Scale (BBS)*: The BBS is a balance assessment comprising 14 components. Each component is scored from 0 (*lowest level of function*) to 4 (*highest level of function*) points, yielding a total score of 56 points. The BBS is a highly reliable assessment with a 95% confidence interval (CI) for its intraclass correlation coefficient ranging from 0.98 to 0.99 [14].
• Secondary outcome
  – *Chula Parkinson Mobility Scale (Chula PMS)*: This scale assesses mobility in PD patients, comprising three main components: bed mobility, chair transfer, and standing and walking. It yields a total score of 80 and demonstrates a high level of validity and reliability [15].
  – *Gait speed*: Gait speed was assessed using a Neurocom Balance Master (Natus Medical Incorporated, Wisconsin, United States). Participants were instructed to walk across a long force plate at a comfortable speed for three trials, and then, the mean walking speed was calculated.
  – *Step length*: Step length was also measured using the Neurocom Balance Master. Participants walked on a long force plate at their comfortable speed for three trials, and the mean step length was then calculated.
  – *Patient satisfaction*: Patient satisfaction was evaluated using a questionnaire provided to participants at the end of the final session.
  – *Feasibility*: Feasibility was assessed by tracking the number of sessions participants completed in the program and by recording complications arising from the rehabilitation program.

### 2.8. Data Analysis and Statistics

Data obtained from participants who dropped out of the study were excluded from the analysis due to the nonrandomized study design. All data analysis was performed using SPSS Statistics Version 23 (IBM Corporation, New York, United States). The normality of baseline continuous data was assessed using the Shapiro–Wilk test and histogram. As these data were normally distributed, they are presented as mean and standard deviation, and then, unpaired *t*‐tests were used to assess differences between the groups. Categorical data are presented as numbers and percentages, with Chi‐square tests used to assess differences between groups. For outcomes, pre‐ and posttreatment comparisons were made using paired *t*‐tests within each group and unpaired *t*‐tests between groups. In addition, effect sizes were calculated using Cohen′s *d* to quantify the magnitude of change. Statistical significance was set at *p* value < 0.05. Satisfaction survey items and feasibility data were collected and are reported descriptively.

## 3. Results

**Figure 2:**
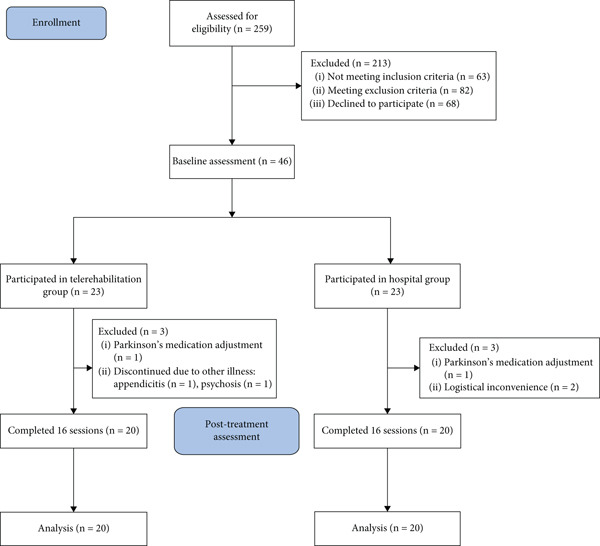
CONSORT flow diagram.

Forty‐six participants were enrolled and divided into two groups: 23 in the tele group and 23 in the hospital group. Three participants in the tele group dropped out due to other illnesses (appendicitis and psychosis) and adjustment to Parkinson′s medication. In the hospital group, two participants dropped out due to an inability to attend at least 80% of the sessions, and one participant withdrew due to an adjustment in Parkinson′s medication (Figure 2). Consequently, 20 participants in each group completed the intervention and were included in the final analysis.

Baseline characteristics and outcome of all participants are shown in Table 1. There were no statistically significant differences between the groups in age, sex, disease duration, modified Hoehn and Yahr stage, BMI, TMSE score, or education level. No statistically significant between‐group differences were observed in baseline BBS scores, Chula PMS scores, gait speed, and step length.

**Table 1:** Demographic data.

	**Telerehabilitation (** **n** = 20**)**	**Hospital (** **n** = 20**)**	**p** **value**
Age, years^a^	69.70 ± 7.48	70.25 ± 6.92	0.615
BMI, kg/m^2a^	21.45 ± 2.36	22.40 ± 3.75	0.346
Duration, years^a^	10.53 ± 7.43	8.28 ± 4.86	0.875
Gender, *n* (%)			1.000
Male	12 (60)	12 (60)	
Female	8 (40)	8 (40)	
Modified Hoehn & Yahr, *n* (%)			0.533
1.5	1 (5)	1 (5)	
2	9 (45)	10 (50)	
2.5	3 (15)	3 (15)	
3	7 (35)	6 (30)	
Level of education, *n* (%)			0.963
Primary	4 (25)	3 (15)	
Secondary	1 (5)	1 (5)	
Bachelor degree	9 (45)	8 (40)	
Postgraduate degree	4 (20)	6 (30)	
TMSE score^a^	27.30 ± 2.00	27.75 ± 1.55	0.432
Baseline BBS score^a^	48.50 ± 4.03	48.55 ± 5.00	0.972
Baseline Chula PMS score^a^	68.60 ± 4.81	69.65 ± 4.95	0.500
Baseline gait speed (cm/s)^a^	70.78 ± 20.49	69.12 ± 20.79	0.843
Baseline step length (cm)^a^	46.58 ± 12.52	45.18 ± 11.12	0.612

Abbreviations: BBS, Berg Balance Scale; BMI, body mass index; Chula PMS, Chula Parkinson Mobility Scale; TMSE, Thai Mental State Examination.

^a^Mean ± SD.

### 3.1. Primary Outcome

There were statistically significant improvements in BBS scores from baseline in both groups (Table 2). The between‐group comparison showed a mean difference of −0.85 (95% CI [−3.12, 1.42], *p* = 0.454) with an effect size (Cohen′s *d*) of −0.24, indicating a small difference (Table 3).

**Table 2:** Effects of telerehabilitation and hospital‐based rehabilitation compared with baseline.

**Outcomes**	**Telerehabilitation (** **n** = 20**)**				**Hospital (** **n** = 20**)**			
	**Mean (SD)**	**p** **value**	**95% CI**	**Intragroup effect size (Cohen**′**s** **d** **)**	**Mean (SD)**	**p** **value**	**95% CI**	**Intragroup effect size (Cohen**′**s** **d** **)**
BBS score								
Pretreatment	48.50 (4.03)				48.55 (4.99)			
Posttreatment	52.00 (3.63)				52.90 (3.35)			
Post–pre	3.50	< 0.001 ^∗^	(2.26, 4.74)	1.32	4.35	< 0.001 ^∗^	(2.35, 6.35)	1.02
Chula PMS score								
Pretreatment	68.60 (4.81)				69.65 (4.95)			
Posttreatment	72.05 (5.26)				75.35 (3.60)			
Post–pre	3.45	< 0.001 ^∗^	(1.69, 5.21)	0.92	5.70	< 0.001 ^∗^	(3.70, 7.70)	1.33
Gait speed (cm/s)								
Pretreatment	70.88 (20.45)				69.56 (21.48)			
Posttreatment	71.45 (20.11)				70.20 (24.81)			
Post–pre	0.57	0.807	(−4.25, 5.39)	0.06	0.64	0.862	(−6.96, 8.24)	0.04
Step length (cm)								
Pretreatment	46.19 (12.55)				44.29 (10.85)			
Posttreatment	47.40 (13.27)				46.96 (11.95)			
Post–pre	1.21	0.610	(−3.68, 6.10)	0.12	2.67	0.177	(−1.31, 6.65)	0.31

Abbreviations: BBS, Berg Balance Scale; Chula PMS, Chula Parkinson Mobility Scale.

∗*p* value < 0.05.

**Table 3:** Effects between telerehabilitation and hospital‐based rehabilitation at posttreatment.

**Outcomes**	**Changes from baseline, mean (SD)**		**Mean difference between groups (tele–hospital)**			**Effect size (Cohen**′**s** **d** **)**
	**Tele (** **n** = 20**)**	**Hospital (** **n** = 20**)**	**Mean (SE)**	**p** **value**	**95% CI**	
BBS score						
Post–pre	3.50 (2.65)	4.35 (4.27)	−0.85 (1.10)	0.454	(−3.12, 1.42)	−0.24
Chula PMS score						
Post–pre	3.45 (3.76)	5.70 (4.28)	−2.25 (1.27)	0.086	(−4.83, 0.33)	−0.56
Gait speed (cm/s)						
Post–pre	0.57 (10.30)	0.64 (16.25)	−0.07 (4.30)	0.987	(−8.78, 8.64)	−0.01
Step length (cm)						
Post–pre	1.21 (10.44)	2.67 (8.51)	−1.46 (3.01)	0.631	(−7.56, 4.64)	−0.15

Abbreviations: BBS, Berg Balance Scale; Chula PMS, Chula Parkinson Mobility Scale; Tele, telerehabilitation group.

### 3.2. Secondary Outcome

Significant improvements in the Chula PMS scores were observed in both groups (Table 2), although the between‐group effect size (Cohen′s *d* = −0.56) indicated a moderate effect (Table 3).

Regarding gait speed and step length, within‐group comparisons revealed no statistically significant differences in either group (Table 2). The between‐group comparison also showed no statistically significant difference with a trivial effect size (Table 3).

Compliance with the rehabilitation program was excellent in both groups. The mean attendance rate was 92.2% (14.75 sessions) in the tele group and 96.5% (15.45 sessions) in the hospital group. Satisfaction rate was also high, with mean scores of 27.7 out of 30 (92.33%) in the tele group and 27.8 out of 30 (92.67%) in the hospital group (Table 4). No adverse events were reported in either group.

**Table 4:** Satisfaction scores in telerehabilitation and hospital groups.

**Satisfaction**	**Scale**	**Mean score in the telerehabilitation group (** **n** = 20 **)**	**Mean score in the hospital group (** **n** = 20 **)**
1. I feel that my information is well‐secured throughout the rehabilitation process.	1–5	4.55	4.65
2. I feel that I can discuss and communicate with physical therapists effectively.	1–5	4.5	4.55
3. I feel that I received highly effective rehabilitation.	1–5	4.55	4.65
4. I feel that the rehabilitation process is convenient and I receive guidance regarding my health issues.	1–5	4.65	4.65
5. I felt free from stress throughout the rehabilitation process.	1–5	4.7	4.6
6. I feel that I have benefited from the rehabilitation.	1–5	4.75	4.8
Overall score (total score of 30), %		27.7, 92.33%	27.8, 92.67%

## 4. Discussion

This study is the first to compare the efficacy of hospital‐based rehabilitation and telerehabilitation in patients with PD using an integrated motion detection system that combines a 3D camera and motion sensors to provide real‐time feedback to both therapists and patients. This system is designed to enhance exercise precision and engagement while enabling remote supervision, addressing a major barrier to accessing regular rehabilitation among individuals with PD.

Previous studies have explored telerehabilitation in PD using various technologies, but most have relied on video‐based monitoring without motion sensor integration. Chen et al. [16] conducted a meta‐analysis on telerehabilitation interventions incorporating virtual reality and found significant improvements in the BBS scores compared to active control interventions. Seidler et al. [17] compared tango dance group therapy with video‐based telerehabilitation and found similar improvements in balance. Cornejo Thumm et al. [18] published a case report involving two individuals with PD who used a virtual reality–based telerehabilitation system that provided real‐time video feedback, resulting in improved gait speed and walking endurance. Similarly, D′Souza et al. [19] implemented a smartphone‐based multimodal telerehabilitation program and achieved good adherence and functional outcomes.

Building on previous approaches, our telerehabilitation system incorporates motion sensor feedback, which may have facilitated more accurate execution of exercises and increased patient confidence during training. These enhanced interactions might have contributed to the comparable improvements observed between the two groups.

Despite statistically significant improvements in BBS scores, these changes did not reach the established minimal clinically important difference (MCID) of 5 points for patients with PD, as reported by Steffen and Seney [20]. This MCID was derived from a population with a wide range of disease severities (Hoehn and Yahr Stages 1–5), whereas our study included only patients with mild to moderate severity of PD, which may partly explain the smaller observed changes. Additionally, some participants had been engaged in exercise programs for more than 2 months prior to enrollment, and any residual benefits from those programs may have limited the potential for further improvement. Nevertheless, the large effect sizes observed in both groups suggest clinically meaningful changes, consistent with previous studies on balance training in individuals with PD [21–23].

Improvements in the Chula PMS were observed in both groups, indicating similar gains in mobility across two rehabilitation settings. Because the MCID for the Chula PMS has not yet been established, the clinical relevance of the within‐ and between‐group difference remains unclear.

In contrast, gait speed and step length did not show significant changes in either group. Variability in gait measures may have been influenced by the use of self‐selected walking speed during testing. Although the intervention primarily focused on step training, it did not specifically target gait mechanics, which may explain the absence of improvement in these parameters. Future programs may benefit from incorporating targeted gait training or wearable gait sensors to enhance the detection and responsiveness of gait‐related outcomes.

In this study, a patient preference trial design was employed to better reflect real‐world conditions. Although this nonrandomized approach introduces potential selection bias, the baseline demographic and clinical characteristics were comparable between the groups, with no statistically significant differences observed. The high satisfaction and attendance rates in the tele group indicate strong acceptability and engagement with this mode of service among patients.

Several challenges were encountered, particularly unstable Internet connections and difficulties using technology among older adults. Pocket Wi‐Fi devices were provided to participants without home Internet access; however, some network instability persisted. Future efforts should focus on improving network reliability and refining the design of telerehabilitation systems to simplify their use and provide a more seamless experience for patients.

Telerehabilitation enhances accessibility by enabling individuals to receive rehabilitation services at home, reducing barriers such as transportation, caregiver dependence, and infection risk. Our findings demonstrate that a motion sensor telerehabilitation system with real‐time feedback can deliver therapeutic outcomes comparable to hospital‐based rehabilitation for patients with mild to moderate PD. The age profile of participants was consistent with the general PD population in Thailand [24], further supporting the external validity of the findings. Overall, our telerehabilitation system represents a practical complement to conventional therapy and a feasible option for patients with limited access to rehabilitation services in hospitals. Despite these promising findings, several limitations should be acknowledged.

### 4.1. Study Limitations

This study has several limitations. First, as a pilot trial using a patient preference design without randomization, there is a potential risk of selection bias. Second, the small sample size limited the statistical power to detect differences between the tele and hospital groups. Third, only short‐term outcomes were assessed, and the long‐term effects of the intervention remain unknown. Fourth, technical challenges, such as unstable Wi‐Fi connection and limited network efficiency, affected the smooth delivery of rehabilitation sessions. Lastly, the study included only patients with mild to moderate motor symptoms (modified Hoehn and Yahr Stages 1.5–3), which limits the generalizability of the findings to more advanced severity.

Further research is needed to evaluate the long‐term effects of this intervention, the feasibility of wider implementation, and its cost‐effectiveness.

## 5. Conclusion

This study indicates that the motion sensor telerehabilitation system produced meaningful improvements in balance and physical function among individuals with mild to moderate PD. Although both intervention and control groups improved, no statistically significant differences were observed between them. The telerehabilitation approach also showed high feasibility and patient satisfaction. However, the improvements in the BBS did not reach the MCID, highlighting the need for continued research. Future investigations should evaluate this system in larger hospital settings and in patients with more advanced PD to determine its broader applicability.

## Conflicts of Interest

The authors declare no conflicts of interest.

## Funding

This research was supported by the Thailand Science Research and Innovation Fund, Chulalongkorn University (grant number: CU_FRB65_hea(31)_038_30_19).

## Data Availability

The data that supports the findings of this study are available from the corresponding author upon reasonable request.
